# Genomic analysis reveals genes affecting distinct phenotypes among different Chinese and western pig breeds

**DOI:** 10.1038/s41598-018-31802-x

**Published:** 2018-09-06

**Authors:** Zhe Zhang, Qian Xiao, Qian-qian Zhang, Hao Sun, Jiu-cheng Chen, Zheng-cao Li, Ming Xue, Pei-pei Ma, Hong-jie Yang, Ning-ying Xu, Qi-shan Wang, Yu-chun Pan

**Affiliations:** 1 Department of Animal Science, School of Agriculture and Biology, Shanghai Jiao Tong University, Shanghai, 200240 P.R. China; 2 Shanghai Key Laboratory of Veterinary Biotechnology, Shanghai, 200240 P.R. China; 3 Animal Genetics, Bioinformatics and Breeding, University of Copenhagen, Frederiksberg, 1870 Denmark; 4 College of Animal Sciences, Zhejiang University, Hangzhou, 310058 P.R. China; 5 National Station of Animal Husbandry, Beijing, 100125 P.R. China

## Abstract

The differences in artificial and natural selection have been some of the factors contributing to phenotypic diversity between Chinese and western pigs. Here, 830 individuals from western and Chinese pig breeds were genotyped using the reduced-representation genotyping method. First, we identified the selection signatures for different pig breeds. By comparing Chinese pigs and western pigs along the first principal component, the growth gene *IGF1R*; the immune genes *IL1R1*, *IL1RL1*, *DUSP10*, *RAC3* and *SWAP70*; the meat quality-related gene *SNORA50* and the olfactory gene *OR1F1* were identified as candidate differentiated targets. Further, along a principal component separating Pudong White pigs from others, a potential causal gene for coat colour (*EDNRB*) was discovered. In addition, the divergent signatures evaluated by *F*_*st*_ within Chinese pig breeds found genes associated with the phenotypic features of coat colour, meat quality and feed efficiency among these indigenous pigs. Second, admixture and genomic introgression analysis were performed. Shan pigs have introgressed genes from Berkshire, Yorkshire and Hongdenglong pigs. The results of introgression mapping showed that this introgression conferred adaption to the local environment and coat colour of Chinese pigs and the superior productivity of western pigs.

## Introduction

Pigs were independently domesticated in Europe and China approximately 9000 years ago^1–4^. Since then, various pig breeds have been subjected to different forces of natural and artificial selection, which have been some of the contributory factors to the distinct phenotypes of different pig breeds^5^. Chinese pig breeds are famous for high prolificacy^6^, good adaptability to local environment^7^, high resistance to disease^8–10^ and desirable meat quality^11,12^. However, there is still variation in these characteristics among different Chinese pig breeds. Compared with Chinese local breeds, European pig breeds are renowned for their fast growth rate^13^, high feed efficiency^14^ and superior meat yield^15^.

Therefore, it is possible to use selection signature detection methods to elucidate the genetic background of the distinct phenotypes of pig breeds, which were influenced by different selection pressures. Moreover, the availability of genomic data facilitates the identification of the genomic regions affecting the specific characteristics among different pig breeds. For instance, by comparing the genomes of Tibetan pigs with low-land pigs, belted and non-belted pigs, Ai *et al*.^16^ discovered *ADAMTS12*, *SIM1* and *NOS1* as candidate genes contributing to high-altitude adaption and *EDNRB* as a gene affecting coat colour^16^. Based on a comparison between Chinese and European pigs, Yang *et al*.^17^ found that the *JAK2* gene was associated with immune response in Chinese pigs and that the *IGF1R* gene was associated with growth in European pigs^17^.

In addition, there has been gene flow from Chinese pigs to European pig breeds since the nineteenth century^18^ aiming to improve the productivity of local breeds. A study has shown that Asian pig haplotypes have been introgressed into European pig breeds to improve traits of commercial interest^19^. For example, reproduction^20^ and carcass and meat quality traits^21^ in European pigs have been improved by the introduction of Asian haplotypes. Conversely, European haplotypes might also have been introgressed into Chinese pig breeds. To obtain the superior characteristics of western pigs, human-mediated hybridization and introgression were performed to improve the productivity and environmental adaptability of Chinese pig breeds. This is similar to the formation of the hybrid nature of Chinese Sutai pigs cultivated from Chinese Erhualian and Duroc pigs^16,22^. However, except the hybrid breed, Sutai, introgression from western pigs into other Asian pigs has not been reported so far.

In this study, we collected samples from western pig breeds and different Chinese pig breeds with distinct phenotypic characteristics in the Yangtze River Delta (YRD) area in China. By comparing the genomes between Chinese and western pig breeds and among Chinese pig breeds, we were able to identify genes associated with the distinct phenotypes in these pig breeds. Because of the history and location of the YRD, it plays an important role in international communication, as well as in gene flow. Some Chinese pig breeds in this region might have gene flow from western breeds.

Therefore, the objective of the study is to (1) identify genes associated with the distinct phenotypic characteristics between western and Chinese pig breeds and within different Chinese pig breeds and (2) characterize the introgression from western into Chinese pig breeds.

## Methods

### Ethics statement

All experimental procedures were approved by the Institutional Animal Care and Use Committee of Shanghai Jiao Tong University, and all methods involving pigs were in accordance with the agreement of the Institutional Animal Care and Use Committee of Shanghai Jiao Tong University (contract no. 2011-0033).

### Populations and Data

**Figure 1:**
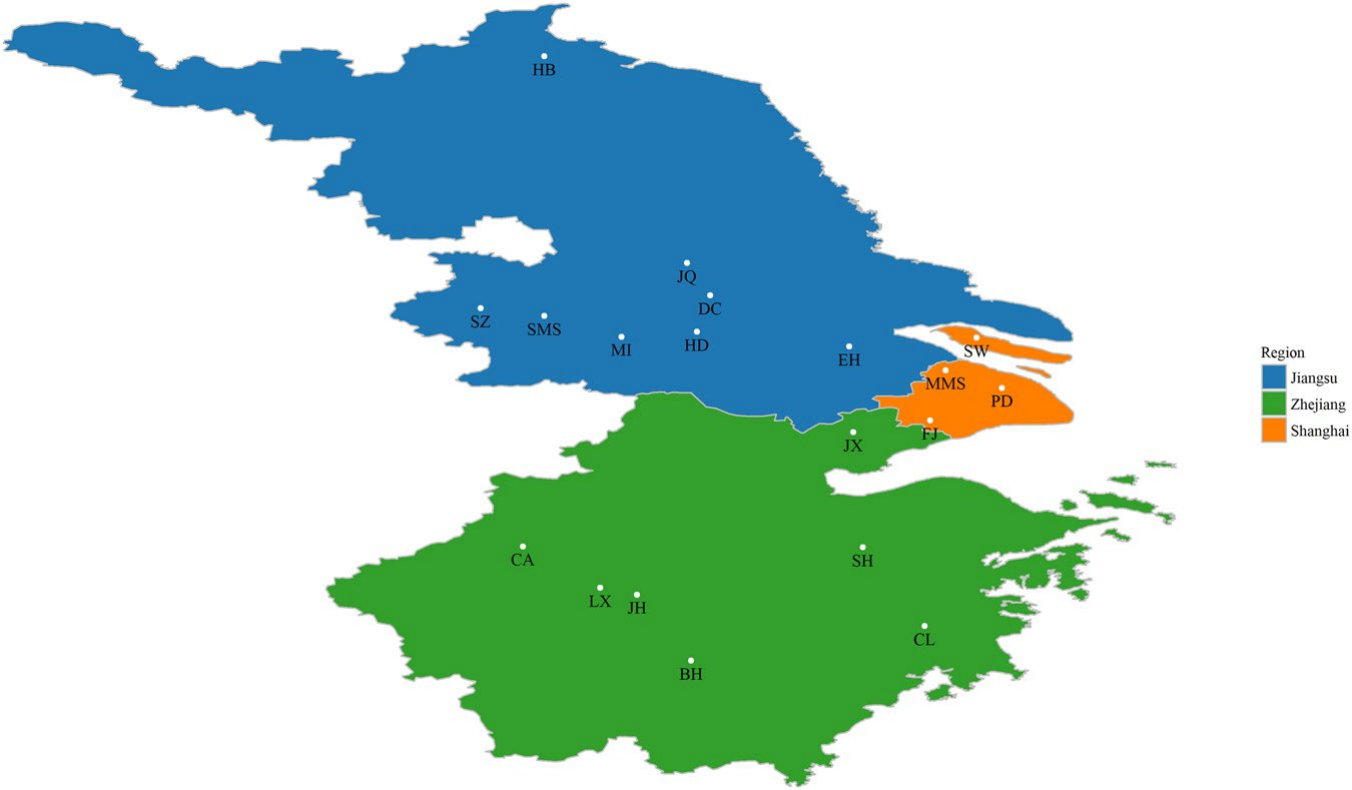
Sample location of Chinese indigenous pig populations within the Yangtze River Delta used in this study. For abbreviations, see Table 1.

A total of 830 pigs were used in this study, including 156 western pigs, i.e., Duroc (D), Landrace (L), Yorkshire (Y), Berkshire (B) and Pietrain (P) pig breeds, and 674 Chinese indigenous pigs within the YRD region (covering Jiangsu and Zhejiang provinces and Shanghai municipality). Figure 1 shows the location where the samples of Chinese pigs were collected. Table 1 lists detailed information of the sampled pig breeds in this study, including breed name, abbreviations and sample size. Most of the reduced-representation genotyping data has been described in previous studies^23–26^, except for that from the Pietrain and Berkshire populations.

**Table 1:** Sample size and allele richness for each population.

Region	Breed	Code	Size	Allele Richness
Western	Duroc	D	48	1.44
	Landrace	L	37	1.48
	Yorkshire	Y	35	1.50
	Pietrain	P	20	1.45
	Berkshire	B	16	1.35
	Small Meishan	SMS	69	1.63
Jiangsu	Mi	MI	36	1.75
	Erhualian	EH	31	1.65
	Dongchuan	DC	8	1.43
	Huaibei	HB	33	1.58
	Hongdenglong	HD	30	1.50
	Jiangquhai	JQ	38	1.73
	Shan	SZ	19	1.73
Zhejiang	Bihu	BH	25	1.56
	Chunan	CA	53	1.68
	Chalu	CL	37	1.60
	Jinhua	JH	53	1.59
	Lanxi	LX	16	1.49
	Shengxian	SH	63	1.67
	Jiangxing Black	JX	29	1.59
Shanghai	Middel Meishan	MMS	50	1.62
	Shawutou	SW	21	1.61
	Fengjing	FJ	16	1.46
	Pudong White	PD	47	1.53

The individuals of the Pietrain and Berkshire populations were genotyped according to the protocol of GGRS (genotyping by genome reducing and sequencing)^27^. In the process of GGRS, genomic DNA was extracted from ear tissue using a commercial kit (Lifefeng Biotech Co., Ltd, Shanghai, China). After the DNA samples were digested with AvaII enzyme and ligated with a unique adapter barcode, the samples were pooled and enriched to construct a sequencing library. DNA libraries (fragment lengths ranging from 300 to 400 bp) were sequenced using an Illumina HiSeq4000 platform according to the manufacturer’s protocol. Quality control of sequences was performed using NGS QC Toolkit^28^ v2.3, and the parameters were set according to the report from Chen *et al*.^27^. Sequencing reads were aligned to the Sscrofa10.2 pig reference genome using BWA^29^.

The BAM files from alignments were used to call SNPs. To improve the precision of SNP detection, SNP calling was performed by both SAMtools^30^ v0.1.9 (set 1) and GATK UnifiedGenotyper^31^ with “hard filters” (QD > 20.0 && FS < 60.0 && MQRankSum > −12.5 && ReadPosRankSum > −8.0) by the VariantFiltration tool (set 2) simultaneously. The SNPs found in both set 1 and set 2 were retained for further steps. Beagle^32^ v4.1 was utilized to impute the missing genotypes in the present study with default parameters. After imputation, SNPs were filtered out if their minor allele frequencies (MAFs) were less than 0.05. Non-autosomal SNPs were also discarded because the demographic patterns of sex chromosomes are different, which may cause distortion in the subsequent analysis^33^. PLINK^34^ v1.07 was used to filter the SNPs with extreme deviations (*p*-value ≤ 1 × 10^−6^) from Hardy-Weinberg equilibrium proportions for each population, and the union set of SNPs that failed to pass the test within at least one population were also discarded. In total, 129,882 high-confidence SNPs were retained for further analysis. Generally, these SNPs were roughly distributed uniformly across the genome, which can represent the information of the whole genome (Supplementary Fig. S1).

### Population structure and genetic diversity

To illustrate the population structure and evaluate the genetic diversity within and between these populations, the following steps were performed: (1) principal component analysis (PCA) was conducted using SMARTPCA integrated in EIGENSOFT^35^ v6.1.4, which transformed the genetic variation into continuous axes (principle components) by singular value decomposition. (2) A total of 41,118 SNPs, which discarded ones that were in LD (linkage disequilibrium) larger than 0.5 across these populations (command: PLINK–indep 50 5 2), were kept for population structure analysis using ADMIXTURE^36^ v1.3.0. The number of ancestral clusters (K) was set from 2 to 40, and five-fold cross-validation was run to determine the K value with the lowest cross-validation error. The result was shown by DISTRUCT^37^ v1.1. (3) The allelic richness was calculated by ADZE^38^ v1.0, which can correct for unequal sample size using a rarefaction procedure^39–41^.

### Effective population size

The historical effective population size (*Ne*) was estimated based on the SNP data used in admixture analysis by the software SNeP^42^ v1.1, which can estimate *Ne* at different *t* generations based on LD between SNPs with the distance of *c*, where $t={(2c)}^{-1}$, and *c* is the distance measured in Morgan^43^ (assuming 100 Mb = 1Morgan). Some options were also used for SNeP software: (1) sample size correction for unphased genotypes; (2) correction to account for mutation; (3) Sved & Feldman’s recombination rate modifier^44^.

### EigenGWAS analysis

Inspired by the PCA result that the first principal component (PC1) clearly separated the Chinese and western pigs, a method called EigenGWAS^45^, which considered PC1 as the phenotype, was used to identify loci associated with the pattern of PC1. Additionally, as the result of the third principal component (PC3) showed, the Pudong White pig population (PD) was obviously separated from the other populations on this axis. Given that PD is a unique Chinese indigenous pig breed covered by a wholly white coat, PC3 was also considered as a phenotype for EigenGWAS analysis. This method corrected for genetic drift by using a genomic inflation factor^46^. After this correction, the *p*-values of SNPs were further corrected by Bonferroni correction, and the cut-off was 0.05/129882. Moreover, in order to validate whether the EigenGWAS analysis with PC1 as the phenotype could identify differentiation between Chinese and western pigs, Weir and Cockerham’s *F*_*st*_^47^ between them was calculated using VCFtools^48^. Finally, the Spearman’s rank correlation coefficient between Weir and Cockerham’s *F*_*st*_ and the negative logarithm of *p*-values of the EigenGWAS with PC1 as the phenotype was calculated in R^49^.

### Identification of selection signatures among Chinese indigenous pigs by *F*_*st*_

To identify highly differentiated genomic regions among Chinese indigenous pig breeds, an *F*_*st*_ outlier approach implemented in the R package OutFLANK^50^ was used to find significant differentiation loci among these pig breeds. Firstly, the near-independent SNPs were identified using R package bigsnpr^51^ according to the tutorial of OutFLANK. Based on this near-independent SNP set, OutFLANK fit a chi-square distribution to the core distribution of *F*_*st*_ (that is, trimming the top and bottom 5%) to estimate the mean and degree of freedom, so that this core distribution would not be affected by strong balancing and diversifying selection. Then the *p*-values of all the SNPs with heterozygosity greater than 0.1 were calculated based on the core distribution of *F*_*st*_. OutFLANK adjusted multiple *p*-values to *q*-values^52^, and the threshold of 0.01 was used^53^.

### Three-population test

To investigate the statistical significance of admixture among these pig populations, TreeMix^54^ software was used to perform the three-population (*f3*) test^55^. In the *f3* test with the form of *f3* (A; B, C), an extreme negative *f3* statistic indicates that significant gene flow to population A from populations B and C exists. All 24 populations were included in the *f3* test, and this would generate $$(\begin{array}{c}24\\ 3\end{array})=6072$$ different combinations. The SNP set that had been LD filtered for the ADMIXTURE analysis was used in this step. A block jackknife^56^ implemented in TreeMix with a window of 200 SNPs that excluded the dependence between different windows was used to calculate the standard deviation of the test. Then, the Z scores were calculated, and combinations that had Z scores less than −2 were regarded as significant.

### Mapping of admixture along the genome using PCAdmix

The results of the *f3* test only showed that the Shan pig (SZ) was significantly admixed by Hongdenglong (HD), B and Y. PCAdmix^57^ v1.0 was used to identify probable significant admixed fragments due to genomic introgression from the three ancestral populations into SZ. Based on PCA, PCAdmix can infer the ancestry of admixed genomes from ancestral individuals using a sliding window along the genome. Then, the posterior probability of ancestry affiliation for each window can be determined by a hidden Markov model. PCAdmix requires phased genotypes, therefore the genotype data used in the ADMIXTURE analysis was phased by fastPHASE^58^ v1.2 with default parameters. According to Barbato *et al*.^59^, the window size was set to be a fixed value of 5 SNPs due to no available linkage map and a low density of markers^59^. PCAdmix inferred the posterior probability (PP) of ancestry from the HD, B and Y populations for each individual haploid genome of SZ for each window. Then, the PP for each reference population was added up across all the haploid genomes of SZ to calculate the scores of affiliation of different ancestry for each window. The windows with the top 1% of scores for each ancestry affiliation were selected to be candidate genomic introgression regions, and genes within these regions were extracted using the biomaRt^60^ package.

### Functional annotation of candidate genes

ANNOVAR^61^ was used to identify candidate SusScr3 Ensembl genes near the significant SNPs based on EigenGWAS and *F*_*st*_ (within 150 kb). Functional gene set enrichment analysis was then performed for these gene sets and candidate genes within the top genomic introgression regions from the PCAdmix analysis. The R package org.Ss.eg.db was used to annotate the pig genes. Enriched Gene Ontology^62^ (GO) terms were then identified using the R package GOstats^63^. Then, the *p*-value of each GO term was calculated by GOstats using a hypergeometric test. The results with a *p*-value ≤ 0.05 were reported to identify potential biological processes influenced by these genes.

## Results and Discussion

### Population structure and genetic diversity

**Figure 2:**
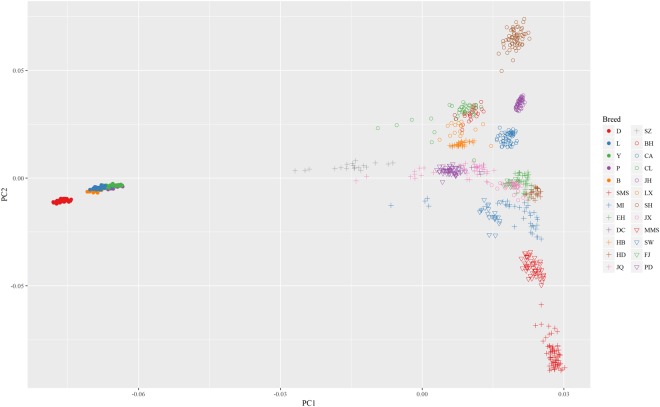
Scatter plot for the first and second principal components. The x-axis represents the first principal component, and the y-axis represents the second principal component. Points of individuals from the same region (Table 1) are the same shape but different colours.

An overview of the relationships among these populations is presented in Fig. 2. PC1, which accounted for 13.0% of the total variance, separated the Chinese and western pigs. Except for Duroc pigs, all individuals from the western pig breeds were clustered together. This is consistent with the breed’s history, since Duroc pigs were developed in the United States, while other western pig breeds were originated from the European continent. Western pig populations were clustered more compactly than those from Chinese pig populations. PC2, which explained 4.0% of the total variance, separated most of Chinese indigenous pig breeds (Fig. 2). The first two components together still could not separate some Chinese pig populations. For example, the points representing the PD pig breed, overlapped with some points of the pig breeds from Jiangsu Province (points in the shape of cross). However, along the third component (PC3), PD was clearly separated from the other pig breeds (Supplementary Fig. S2). The allelic richness results (Table 1) reflected that more genetic diversity existed within Chinese pig populations. Chinese pig populations had more allelic richness compared with western pig populations, except for those in Dongchuan (DC), Fengjing (FJ) and Lanxi (LX), with sample sizes of 16 or less (Table 1). This is due to the tendency for populations with lower sample sizes to have fewer distinct alleles, although ADZE can correct for sample size.

**Figure 3:**
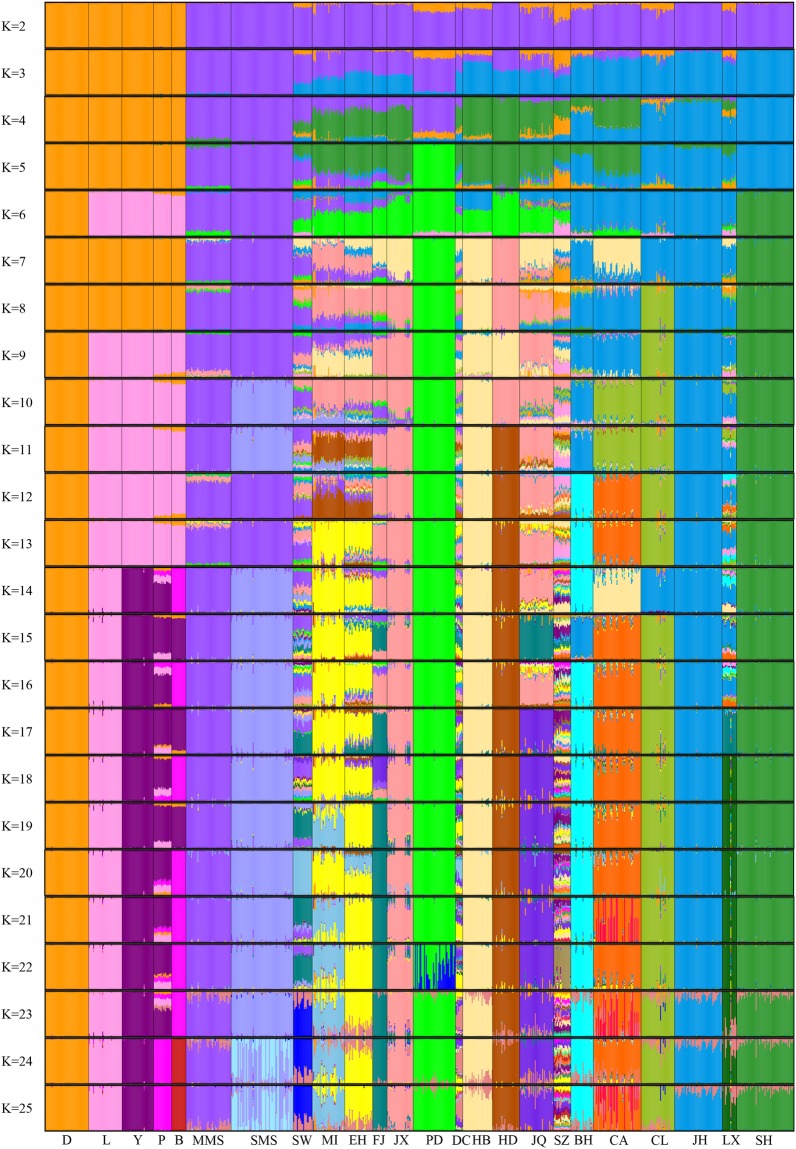
Admixture plot comprising ancestry numbers (K) from 2 to 25 of all the individuals analysed in this study. The estimated K is 25.

The estimated K for the ADMIXTURE analysis with the lowest cross-validation error was 25, nearly the same as the number of actual populations in this study. When two ancestors were assumed, Chinese indigenous pigs and western pigs were clearly distinguished (Fig. 3), but some Chinese pig populations contained some genetic ancestry that was similar to western populations, especially for SZ, which was always the most admixed population over different K values. Compared with a previous study^22^ which exhibited approximately 20% Chinese admixture in most European breeds (with K = 2) using 60 K porcine SNP array, no such much admixture could be observed in this study. This might be due to ascertainment bias resulting from the development of the SNP array mainly based on the polymorphisms distributed in western pig breeds, which would overestimate the shared ancestry between Chinese and western pig breeds. When K was 25, all of the populations roughly had their own ancestry, except for Small Meishan (SMS), Chunan (CA) and SZ. SMS and CA contained new ancestry, which might not be part of the populations included in this study. More pig populations are needed to identify the distinct genetic components not shared with other breeds in the study. Compared with Chinese pigs, the extents of admixture within western pig populations across different K values were low and stable.

### Effective population size

The estimation of *Ne* of each pig breed across the generations is shown in Supplementary Fig. S3. The past *Ne* was reflected by LD over shorter recombination distances, and the longer distances provided recent *Ne*^43^. From 900 generations to approximately 50 generations ago, all the breeds exhibited a decrease in *Ne* estimates over time. However, between 900 and 1000 generations ago, there were some obvious inflection points in several lines. The nearest anti-climax point indicated the nearest starting point of artificial selection, which caused the bottleneck in the population^64^. In general, the *Ne* of western pig breeds was smaller than that of Chinese pig populations, which was due to the higher LD of western pig breeds^65^. Admixture is a potential confounding factor for the estimation of *Ne* that can cause bias^66^. Therefore, among Chinese indigenous pigs, the populations with a low extent of admixture tend to have a smaller *Ne*^67^. The *Ne* estimates did not get stable even in recent generations. This sort of trend could also be found in another study for Landrace and Yorkshire pigs^68^. For western pigs, the reason of this phenomenon might be the ongoing strong selection on production traits^5^, whereas for Chinese local pig breeds, it might be due to the inbreeding caused by small population size^69^.

### EigenGWAS for PC1 and PC3

**Figure 4:**
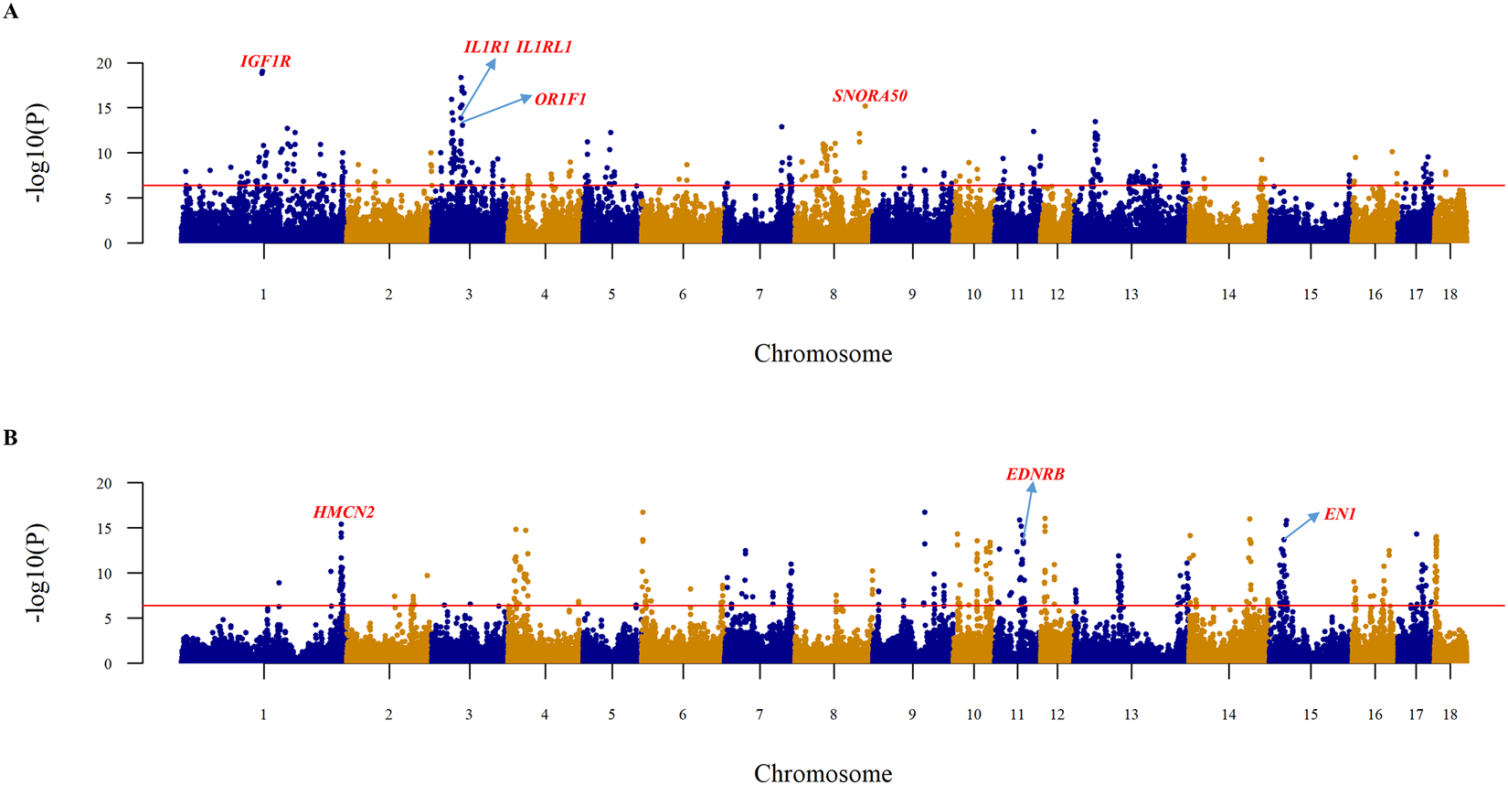
Manhattan plot of EigenGWAS. The x-axis represents the locations of SNPs, and the y-axis represents the negative logarithm of the *p*-values for the EigenGWAS that used PC1 (**A**) and PC3 (**B**) as the phenotype, respectively. The red line represents the threshold for statistical significance.

To find loci that were related to the pattern of PC1, which separated the western and Chinese pigs, EigenGWAS with PC1 as the phenotype was performed. Further, this association study was also performed to explain why PD was separated from the other populations on PC3. The Manhattan plots for PC1 and PC3 are shown in Fig. 4A,B, respectively. There were as many as 353 and 414 significant SNPs associated with PC1 and PC3, respectively. EigenGWAS aims to find ancestry informative markers (AIMs), which can be found in huge quantities if the genetic backgrounds of populations are very different. The Spearman’s correlation coefficient between Weir and Cockerham’s *F*_*st*_ and the negative logarithm of *p*-values of the EigenGWAS with PC1 as the phenotype was 0.969. Some studies have suggested that individual-level eigenvectors are measures of population differentiation reflecting *F*_*st*_ among subpopulations^35,70,71^. Therefore, the high value of this correlation coefficient validated that the EigenGWAS with PC1 as the phenotype could reflect the differentiation between Chinese and western pigs. In this study, it is difficult to determine whether these differentiated signals were formed in the pre-domestication or during the divergent post-domestication selection. However, both of these different kinds of significant signals could help explain the genetic background of distinct phenotypes between Chinese and western pigs. In addition, the genomic inflation factors were 100.81 and 17.75 for the EigenGWAS with PC1 and PC3 as the phenotype, respectively. According to the original paper on EigenGWAS^45^, the genomic inflation factors are highly correlated to eigenvalues, which were 107.58 and 24.26 for PC1 and PC3, respectively. A large eigenvalue indicates underlying population structure. Therefore, correction for genomic inflation factors will filter out signals due to population stratification, allowing loci under selection to be identified^45^.

There were 286 Ensembl genes located near the significant SNPs of the EigenGWAS for PC1 (Supplementary Table S1). The first and second significant SNPs on chromosome 1 were located near and within the *IGF1R* gene. This gene plays an important role in pig production traits, such as post-natal growth^72^ and carcass and meat content^73^. In addition, a previous study verified that different alleles of the gene *IGF1R* are highly correlated with pig performance based on litter size^74^, indicating that pleiotropy of the *IGF1R* gene can be a potential explanation of the genetic relationship between traits of production and reproduction^75^. Therefore, the differentiated variants in the *IGF1R* gene may be considered a potential quantitative trait locus (QTL), which can account for the phenotypic differences of growth and reproduction between Chinese and western pig breeds. Besides, Chinese pigs are well adapted to their local environments^7^ and are known for their desirable meat quality^11,12^. These phenotypic characteristics could be explained by genes near significant signals on different chromosomes (Fig. 4A). Among them was the olfactory gene, *OR1F1*. A sharp sense of smell is very important for pigs to improve their appetite for roughage feed, and may also help increase their preference for specific food to produce human-desired meat under captivity^76^. In addition, the gene *SNORA50*, located near the top significant SNP on chromosome 8, was identified as a candidate gene for meat quality in a previous GWAS study^12^. Two immunity-related genes, *IL1RL1* and *IL1R1*, were identified on chromosome 3. *IL1R1* is a mediator gene involved in many cytokine-induced immune and inflammatory responses. *IL1RL1* plays an important role in some human diseases such as rheumatoid arthritis and asthma^77^. In human, asthma is a counterpart disease of swine mycoplasmal pneumonia. Chinese indigenous pigs, especially pigs in the YRD, are very sensitive to *Mycoplasma hyopneumoniae*^78–80^. Intriguingly, the most significant GO term (Supplementary Table S2) was “regulation of respiratory burst (GO:0060263)”, which might be related to Mycoplasmal pneumonia. In addition to respiration-related terms, there were also some mast cell-related GO terms at the top of the list, such as “regulation of mast cell chemotaxis (GO:0060753)”, “mast cell chemotaxis (GO:0002551)” and “mast cell migration (GO:0097531)”. Mast cells are found to participate in the early recognition of pathogens, which plays an important role in immunity^81^. These mast cell- or respiratory-related terms were all enriched by the genes *DUSP10*, *RAC3* and *SWAP70*, together with *IL1R1* and *IL1RL1*, which might explain the high resistance to disease in Chinese pigs^8–10^.

The appearance of the PD pig breed is very distinct from that of other Chinese indigenous pigs due to its wholly white coat. Chinese indigenous pigs are often black and sometimes belted or spotted, but never wholly white like PD pigs. Along PC3, PD pigs were separated from the other populations. Performing EigenGWAS with PC3 as the phenotype might therefore help to explain some particular characteristics of PD pigs, such as its uniqueness of coat colour. The coat colour-related gene *EDNRB*^82^ was identified near significant signals on chromosome 11 (Supplementary Table S3). In a previous study about Chinese raccoon dogs, a SNP in *EDNRB* gene was identified as the causal variant for the determinant of white colour in this animal^83^. Therefore, the identification of this gene might help account for the distinct coat colour of PD pigs. Except for some general biological processes, the enriched GO terms were mainly related to pigmentation (“pigmentation (GO:0043473)” and “pigment cell differentiation (GO:0050931)”), some behaviour- and cognition-related processes (“adult locomotory behavior (GO:0008344)”, “learning (GO:0007612)” and “cognition (GO:0050890)”) and nervous system-related functions (“nervous system process (GO:0050877)”, “neuron apoptotic process (GO:0051402)” and “neuron death (GO:0043524)”) (Supplementary Table S4). PD has long been suspected to be formed by admixture between Chinese and western pig breeds because of its white coat colour. The suspicion of its admixture origin could be overturned by the facts that it belonged to the Chinese indigenous pig cluster along the PC1 axis and that there was no evident admixture from western pigs when K = 25 in the ADMIXTURE analysis.

### *F*_*st*_ among Chinese indigenous pigs

**Figure 5:**
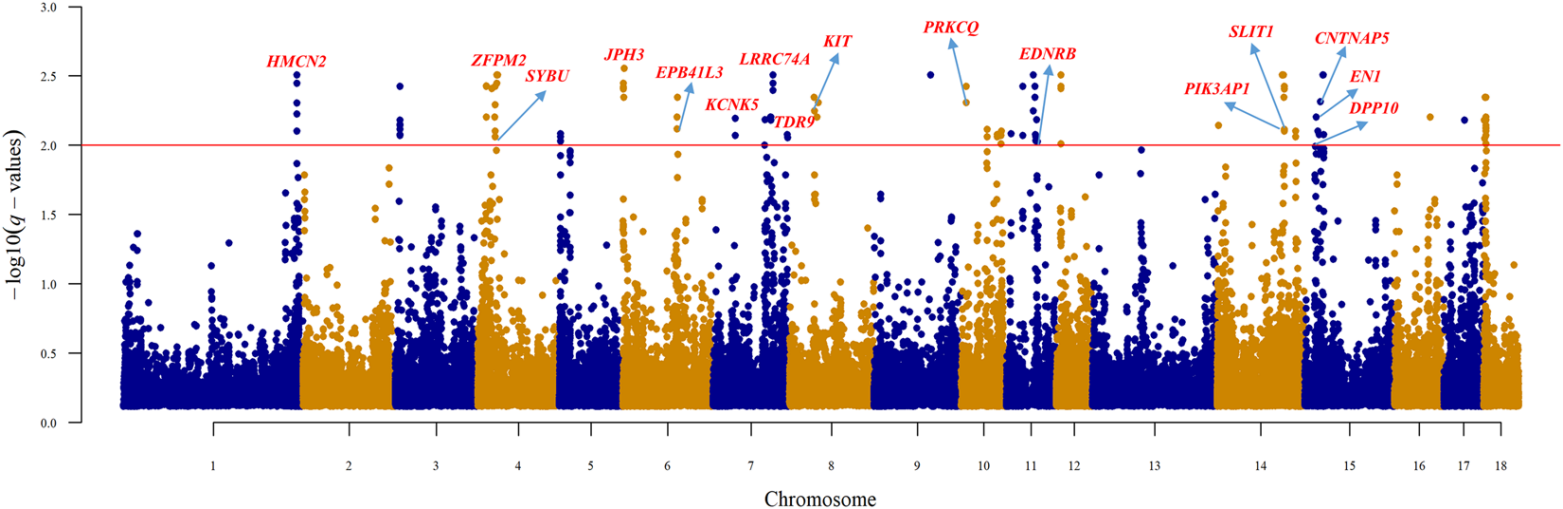
Manhattan plot of *F*_*st*_ for Chinese indigenous pigs. The x-axis represents the locations of SNPs, and the y-axis represents the negative log *q*-values for the *F*_*st*_ values. The red line represents the threshold for statistical significance.

The core distribution of *F*_*st*_ based on 42,746 near-independent SNPs were shown in Supplementary Fig. S4. The estimated mean of the fitted chi-square distribution was 0.23, and the degree of freedom was 14.24. The mean value was not high, indicating that there was not extreme differentiation among Chinese indigenous pigs. The Manhattan plot of *q*-values of 109, 451 SNPs with heterozygosity greater than 0.1 is shown in Fig. 5. After correcting for multiple-testing, there were 129 SNPs identified as significant (Fig. 5). Table 2 lists all the *F*_*st*_ candidate genes that have been verified to be related to pig traits by other studies.

**Table 2:** Candidate genes near significant *F*_*st*_ signals among Chinese pigs that have been verified by previous studies.

Gene	Trait	Reference
*JPH3*	Meat quality	Jeong, *et al*.^86^
*CNTNAP5*	Carcass and meat quality	Rohrer, *et al*.^88^
ENSSSCG00000010493	Cognition	Schachtschneider, *et al*.^93^
*ZFPM2*	Scrotal hernias	Zhao, *et al*.^87^
*HMCN2*	Response to stimulus	Zhu, *et al*.^76^
*LRRC74A*	Teat number	Tan, *et al*.^94^
ENSSSCG00000024537	Lean production	Yu, *et al*.^95^
ENSSSCG00000015700	Lean production	Yang, *et al*.^73^
*EN1*	Development	Ayuso, *et al*.^96^
*SLIT1*	Growth and fatness	Borowska, *et al*.^97^
*PIK3AP1*	Backfat thickness	Zambonelli, *et al*.^98^
*KIT*	Coat color	Moller, *et al*.^89^
*EPB41L3*	Feed efficiency	Reyer, *et al*.^99^
*KCNK5*	Birth weight	Wang, *et al*.^100^
ENSSSCG00000026652	Lean production	Yang, *et al*.^73^
*SYBU*	Meat quality	Chung, *et al*.^101^
*EDNRB*	Coat color	Ai, *et al*.^16^
*TDRD9*	Conformation traits	Le, *et al*.^102^
*DPP10*	Stillbirth	Schneider, *et al*.^103^
ENSSSCG00000016409	Growth	Yang, *et al*.^73^
ENSSSCG00000009338	Feed efficiency	Do, *et al*.^104^
*PRKCQ*	Feed efficiency	Bai, *et al*.^105^

Unlike the EigenGWAS of PC1, which could identify common features of Chinese pigs through the comparison with western pig breeds, *F*_*st*_ signal detection within Chinese pig breeds enabled us to find the differentiated features among these breeds. A total of 75 genes were identified near significant *F*_*st*_ signals (Supplementary Table S5). The first four significant SNPs were all located near the *JPH3* gene. *JPH3* was identified to be associated with boar taint by affecting skatole levels^84^. A previous study verified that some Chinese pigs, such as JH pigs, had a significantly lower level of skatole than Landrace pigs^85^. Boar taint can affect the flavour of pork, which is important in Chinese cuisine. In addition, in another study, this gene was also identified as a candidate target of meat quality traits^86^. Chinese indigenous pigs, such as FJ, JH and DC pigs in this study, are well known for their desirable meat quality and flavor; thus, the *JPH3* gene might have undergone selection to improve meat quality by reducing skatole levels. The most significant signal on chromosome 4 was located in the *ZFPM2* gene. This gene is important in the development of diaphragmatic hernia and a previous study has found it to be significantly associated with pig scrotal hernias^87^. The second significant SNP on chromosome 15 was near *CNTNAP5* gene, which was identified as a candidate gene of pig vertebra number in a previous study^88^. Vertebra number is a trait associated with carcass and meat production. Chinese pigs perform well in the vertebra number trait, and western pigs have also benefited from Chinese pigs in this trait by introgression^21^. The first and second significant signals on chromosome 1 were both located in the *HMCN2* gene, which is related to stimulus response in Chinese pig breeds^76^. As expected, given many pig breeds covered with different kinds of coloured coats, some pigmentation-related genes, such as *KIT* and *EDNRB*, were also identified^89^. In terms of GO enrichment analysis (Supplementary Table S6), the most significant terms were related to pigmentation, such as “pigmentation (GO:0043473)”, “melanocyte differentiation (GO:0030318)” and “pigment cell differentiation (GO:0050931)”. Some GO terms were related to behaviour, such as “locomotion (GO:004001)”. Chinese pigs often have low locomotion and low behavioural reactivity^90^. A previous study has also shown that Chinese pigs have become timid and tame due to selection pressure on behavioural traits during the long time of domestication^76^.

### Three population test and PCAdmix analysis for SZ population

There were only two extreme Z scores for the *f3 test*: −2.23 and −4.62 for the combination of (SZ; Y, HD) and (SZ; B, HD), respectively. This result is consistent with the admixture of SZ (Fig. 3). The admixture might be deliberately human mediated to improve productivity and adaptability to the environment. Mapping the specific regions of admixture can help understand breeders’ agronomic interests as well as the direction of natural selection. Therefore, the PCAdmix analysis was performed to localize the potential regions of gene introgression. The top potential introgression regions along the SZ genome from each of three breeds are listed in Supplementary Table S7. Based on the GO enrichment results enriched by genes introgressed from Y (Supplementary Table S8), the top terms were mainly related to growth (“negative regulation of cell growth (GO:0030308)”, “negative regulation of growth GO:0045926”) and bone development (“*BMP* signaling pathway (GO:0030509)” and “regulation of ossification (GO:0030278)”). Several of the top GO processes enriched by genes introgressed from B (Supplementary Table S9) were related to growth and development, such as “heart development (GO:000750)”and “biomineral tissue development (GO:0031214)”, and some were related to response to external stimulus (“cellular response to corticosteroid stimulus (GO:0071384)”, “cellular response to glucocorticoid stimulus (GO:0071385)” and “sensory perception of pain (GO:0019233)”). The second significant GO term enriched by genes introgressed from HD (Supplementary Table S10) was “toll-like receptor 4 signaling pathway (GO:0034142)”, which is related to immunity. Like the coat colour-related findings mentioned above, another two melanin metabolism processes (“regulation of melanin biosynthetic process (GO:0048021)” and “positive regulation of melanin biosynthetic process (GO:0048023)”) were identified, and *ASIP* was found to be the causal gene. This gene was identified to be related to human pigmentation diversity^91,92^. Given these results, it can be summarized that the introgression regions from western pigs were mainly related to growth and development. On the other hand, the major introgression regions from Chinese pig breeds were related to immunity and pigmentation. Breeders in China preferred the high adaptability and black coat colour of Chinese pigs, while western pigs were chosen for their high productivity. Given the hypothesis that these introgressions were deliberately human mediated, the breeding goal of making good use of characteristics of Chinese and western pigs helped explain these results. Moreover, these results contributed to explaining the genetic basis of the phenotypic distinctions between Chinese and western pigs and within different Chinese pigs.

In this study, the genetic basis of phenotypic differences between Chinese and western pig breeds was studied from the viewpoint of selection signal detection. Numerous genes related to growth, immunity, reproduction and meat quality were identified as candidate differentiated genes, which might contribute to the distinct phenotypes of western and Chinese pigs. In addition, the coat colour-related gene *EDNRB* was identified as a candidate gene for the white colour of the PD pig breed. The significant divergent genetic signals among these Chinese pig populations were related to various economically important traits. Based on admixture and genomic introgression analysis, we observed that there was introgression from western pigs and other Chinese pigs into SZ pigs. The mapping of the introgression also helped to elucidate the genetic basis of phenotypic features, namely, that western pigs are good at production traits, while Chinese pigs do well in adaption to their environments.

## Electronic supplementary material

Supplementary Information

Table S1

Table S2

Table S3

Table S4

Table S5

Table S6

Table S7

Table S8

Table S9

Table S10

## Data Availability

All BAM data were deposited in the National Center for Biotechnology Information (NCBI) Sequence Read Archive (SRA). 264 samples are available under the Bioproject number PRJNA436152. 128 samples are available under Bioproject number PRJNA281578. 438 samples are available under the Bioproject number PRJNA471328.
