# Supplementary material for: Genomic analysis reveals genes affecting distinct phenotypes among different Chinese and western pig breeds
Source: Sci Rep. 2018 Sep 6;8:13352. doi: 10.1038/s41598-018-31802-x (PMC6127261; doi:10.1038/s41598-018-31802-x)
Supplement: Supplementary file 1 — Supplementary Information [file 41598_2018_31802_MOESM1_ESM.docx]

**Genomic analysis reveals genes affecting distinct phenotypes among different Chinese and western pig breeds**

Zhe Zhang^1,2^, Qian Xiao^1,2^, Qian-qian Zhang^3^, Hao Sun^1,2^, Jiu-cheng Chen^4^, Zheng-cao Li^4^, Ming Xue^5^, Pei-pei Ma^1,2^, Hong-jie Yang^5^, Ning-ying Xu^4^, Qi-shan Wang^1,2^ and Yu-chun Pan^1,2^

^1^Department of Animal Science, School of Agriculture and Biology, Shanghai Jiao Tong University, Shanghai 200240, PR China; ^2^Shanghai Key Laboratory of Veterinary Biotechnology, Shanghai 200240, PR China; ^3^Animal Genetics, Bioinformatics and Breeding, University of Copenhagen, Frederiksberg 1870, Denmark; ^4^College of Animal Sciences, Zhejiang University, Hangzhou 310058, PR China; ^5^National Station of Animal Husbandry, Beijing 100125, PR China

Correspondence and request for materials should be addressed to Q.S.W (email: wangqishan@sjtu.edu.cn) and Y.C.P (email: panyuchun1963@aliyun.com)

**Supplementary Figure S1** The distribution of the single nucleotide polymorphisms (SNPs) across the chromosomes. The x-axis represents the chromosome position (Mb), and the y-axis denotes the chromosomes. The number of SNPs present in each 400 kb genome block is illustrated via colours as indicated by the legend.


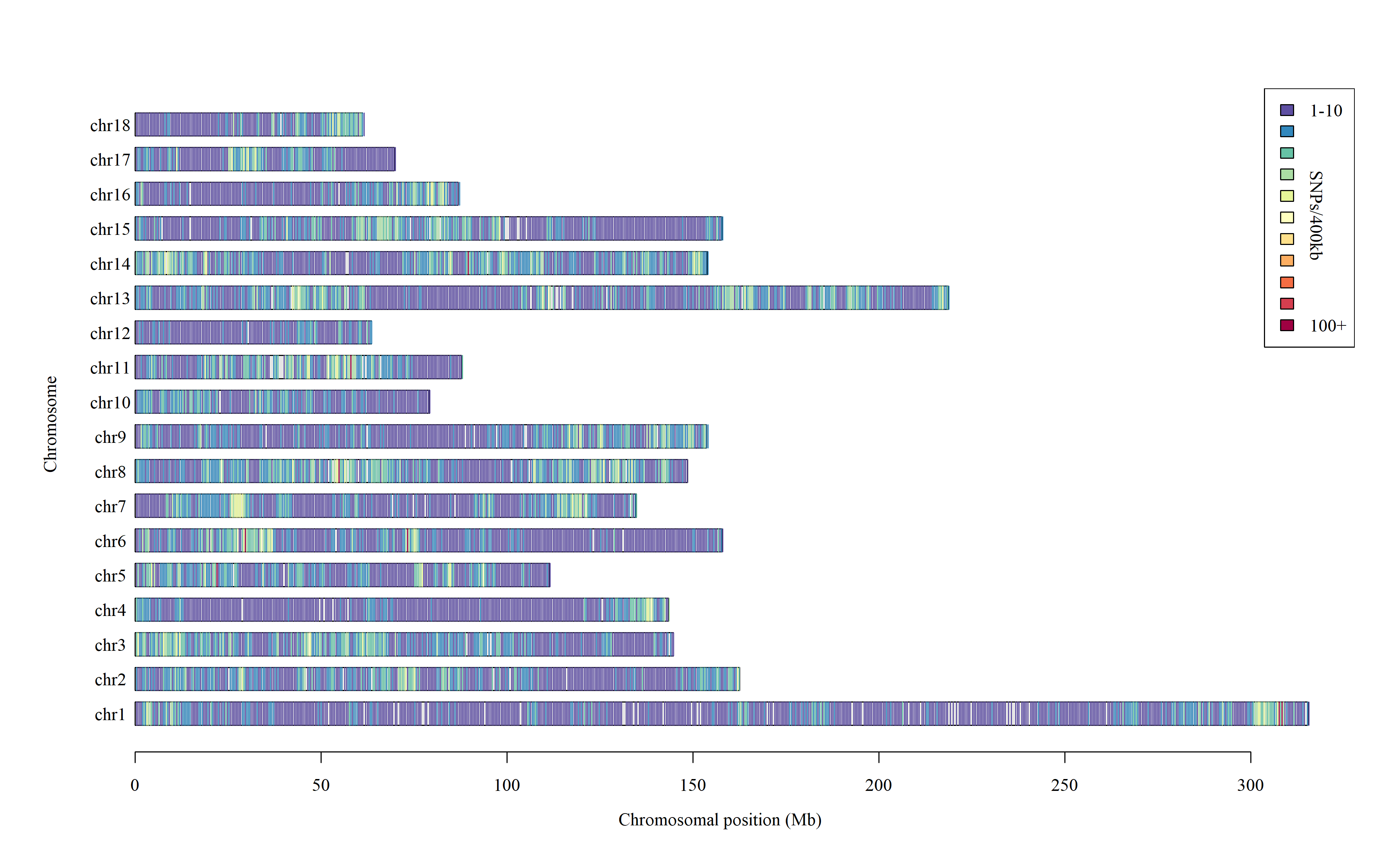


**Supplementary Figure S2** Scatter plot for the second and third principal components. X-axis represents the second principal component and y-axis represents the third principal component. Points of individuals from same region (Table 1) are in same shape but different colors.


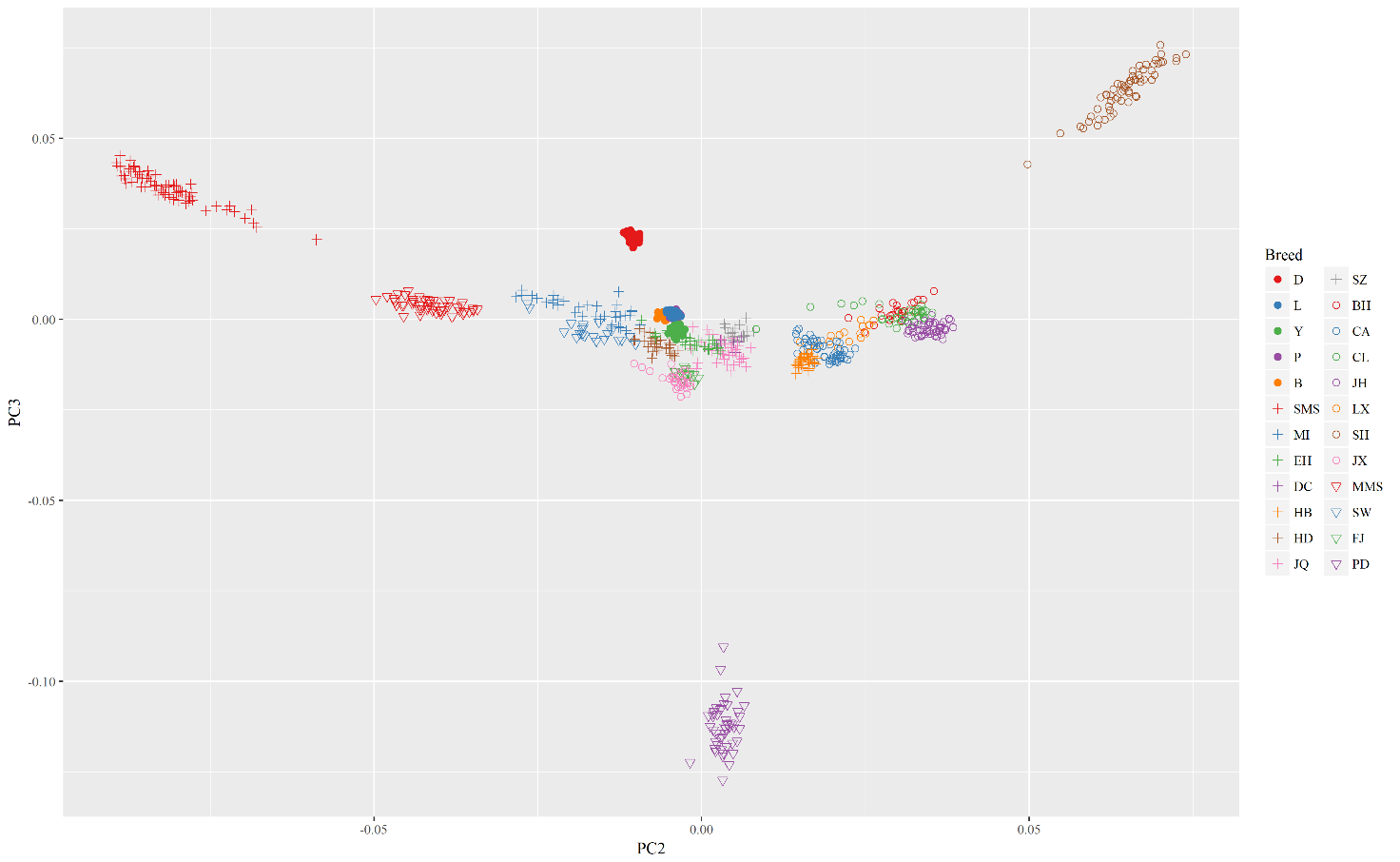


**Supplementary Figure S3** Estimated effective population size (Ne) across generations for each population. Each line shows the trend in effective population size for a pig breed between ~50 and ~1000 generations ago.


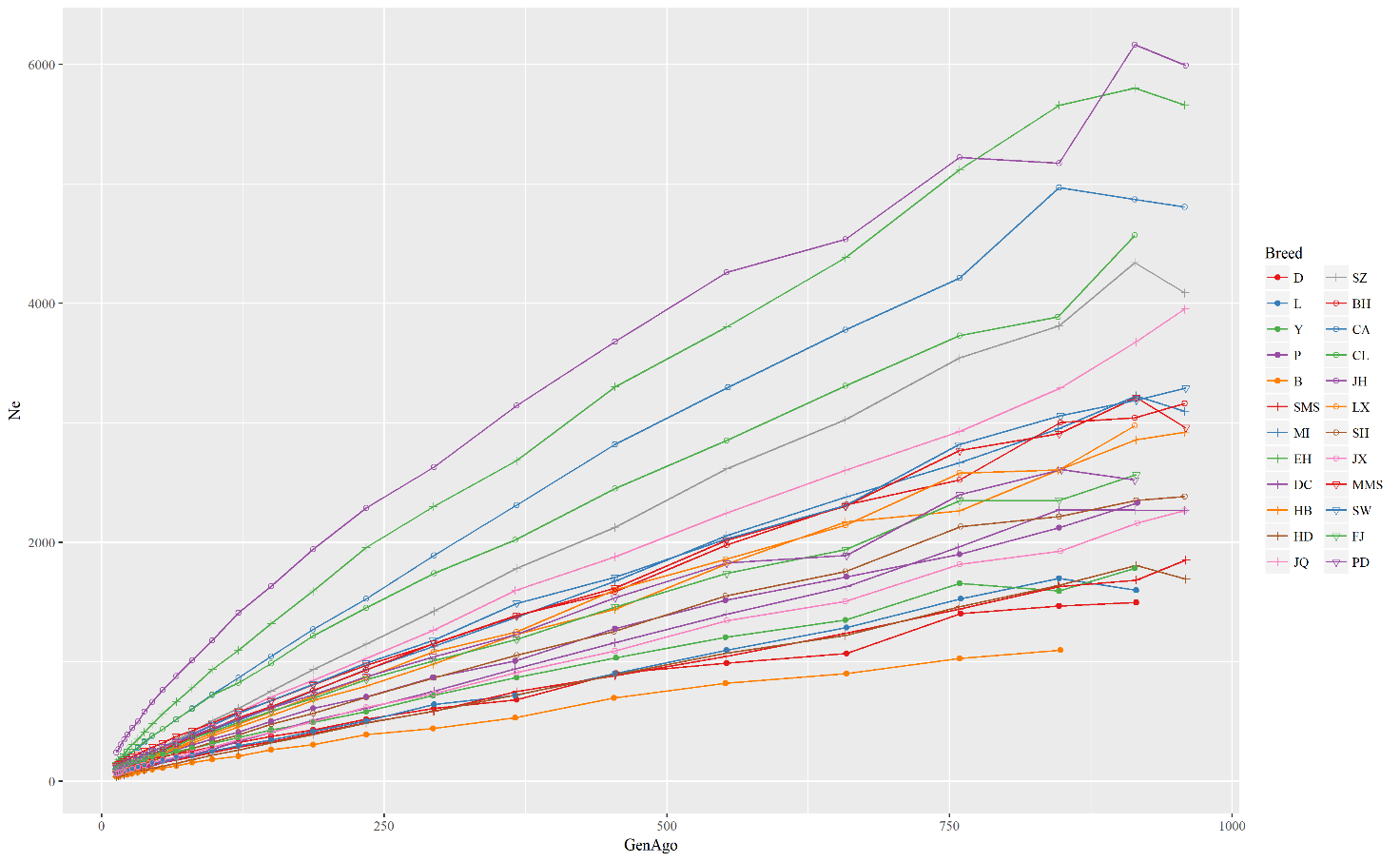


**Supplementary Figure S4** The core distribution of Fst based on the nearly independent SNPs. The blue line indicates the fitted chi-square distribution.

**
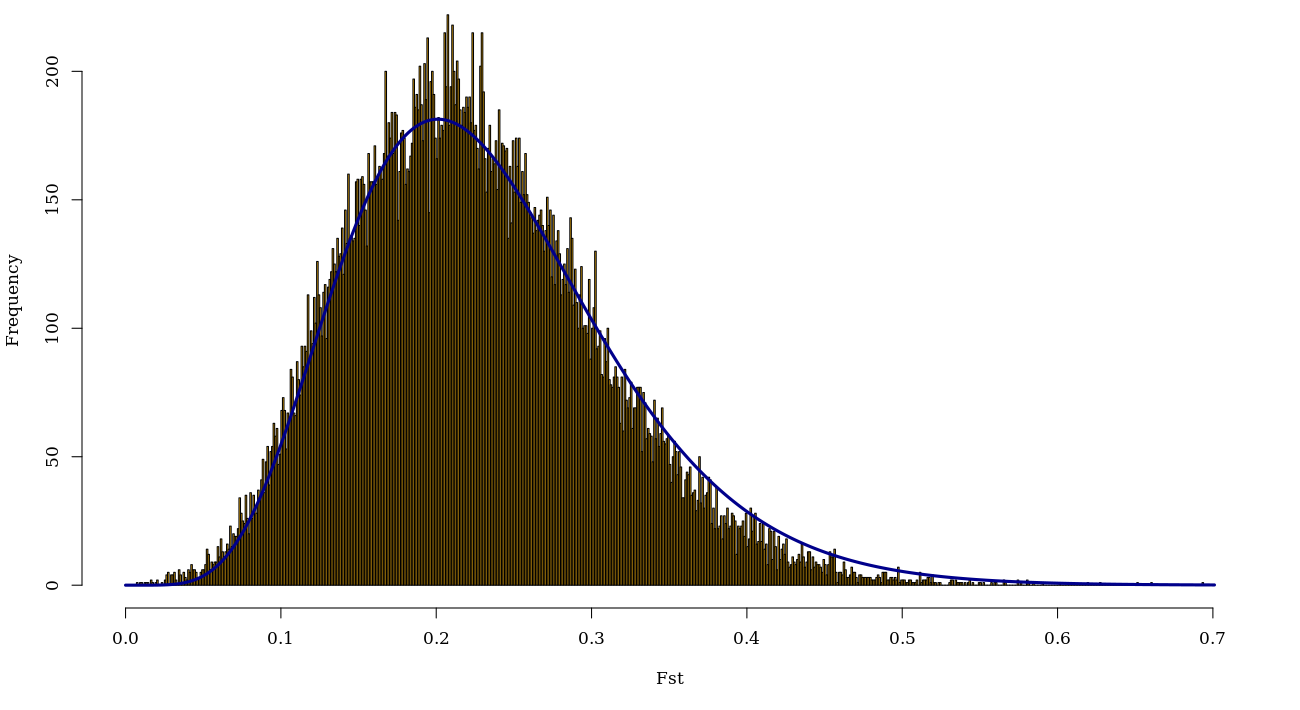
**
